# Suicidal Ideations and Attempts Among Adolescents in Kampala Urban Settlements in Uganda: A Case Study of Adolescents Receiving Care From the Uganda Youth Development Link

**DOI:** 10.3389/fsoc.2021.646854

**Published:** 2021-07-21

**Authors:** Paul Bukuluki, Symon Wandiembe, Peter Kisaakye, Samuel Besigwa, Rogers Kasirye

**Affiliations:** ^1^Department of Social Work and Social Administration, School of Social Sciences, Makerere University, Kampala, Uganda; ^2^Department of Statistical Methods, School of Statistics and Planning, Makerere University, Kampala, Uganda; ^3^Department of Population Studies, School of Statistics and Planning, Makerere University, Kampala, Uganda; ^4^Department of Programmes and Research, Uganda Youth Development Link, Kampala, Uganda

**Keywords:** suicidal ideations, adolescents, urban areas, Kampala, COVID-19

## Abstract

There is an increasing recognition that suicidal ideation is a major public health concern in sub-Saharan Africa. We employed a case study design, taking a case study of adolescents currently under the care of Uganda Youth Development Link (UYDEL). The data analyzed were collected from 219 female and male adolescents (13–19 years) recruited through UYDEL in Kampala, Uganda. A Poisson regression model with robust variance was used to assess the risk factors associated with suicidality. The prevalence of suicidal ideation in the past 4 weeks and attempt within the past 6 months among adolescents was 30.6% (95% CI: 24.8, 38.0%) and 24.2% (95% CI: 18.7, 30.4%), respectively. The most stressful precursors of suicidal ideation or attempt included financial difficulties (59.5%), family breakdown or conflicts (37.4%), and trauma (23.1%). Suicidal ideation in the past 1 week preceding the survey was as high as 13.3% (95% CI: 9.0, 18.6%), of which 75.0% (95% CI: 55.1%, 89.3%) had a suicide plan. Prevalence of suicidal ideation in the past 4 weeks was significantly higher among respondents with moderate psychological distress [Prevalence Ratio (PRR) = 2.74; 95% CI: 0.96, 7.84] and severe psychological distress (PRR = 4.75; 95% CI: 1.72, 13.08) but lower among adolescents who knew where to obtain professional psychological care (PRR = 0.51; 95% CI: 0.30, 0.87). Similarly, suicidal attempt was significantly higher among respondents with moderate psychological distress (PRR = 4.72; 95% CI: 1.01, 12.03) and severe psychological distress (PRR = 11.8; 95% CI: 4.66, 32.37), and who abuse drugs or substances (PRR = 2.13; 95% CI: 1.13, 4.01). Therefore, suicidal ideation is a major public health issue among adolescents living in poor urban settlements in Kampala, Uganda. Psychological distress due to financial difficulties, unemployment, and family breakdown are major facilitators of suicidality among adolescents in urban poor settlements in Kampala. Interventions aimed at preventing suicide among vulnerable adolescents in urban settlements in Kampala, Uganda should incorporate this unique risk factor profile.

## Introduction

Suicide is a major public health concern (Luxton et al., 2012) and one of the leading causes of death among adolescents ages 15–19 years (Hottes et al., 2015; Gjertsen et al., 2019; WHO, 2020a). There is also an increasing recognition that suicidal ideation is a major public health concern in sub-Saharan Africa (SSA) (Page and West, 2011; Quarshie et al., 2020). A few studies in Uganda have indicated that suicidal ideation among youth in Uganda is high in rural areas (21.6%) (Rudatsikira et al., 2007). Other studies (Culbreth et al., 2018, 2021) have reported suicide ideation to be 24% among young people living in urban areas of Uganda. Suicidal ideation and attempts are likely higher among youth living in the slums or on the streets due to the severe psychosocial challenges they face (Swahn et al., 2012). Furthermore, the emergence of COVID-19 could have exacerbated psychological challenges (Bryan et al., 2020; Mamun and Griffiths, 2020; Sher, 2020; Tasnim et al., 2020; Ammerman et al., 2021). This study hypothesized that suicidal ideations and attempts among vulnerable urban youth receiving support from the Uganda Youth Development Link (UYDEL) could be experiencing higher rates of suicidal ideation and attempts than their youth counterparts.

The literature highlights a number of factors responsible for suicide ideation and attempt among adolescents. Suicide ideation or attempt among adolescents may be triggered by factors such as being homeless, poor, or suffering from a particular disease, psychological stress among others (Kessler et al., 2003; Rukundo et al., 2016; Ssebunnya et al., 2019; Nyundo et al., 2020). As Swahn et al. (2012) observe, psychological stress may be caused by death of a loved one, history of disease, discrimination, and being lonely, among others (Asiki et al., 2011; Carr, 2012; Mugisha et al., 2016). The situation can be worsened among adolescents living in urban areas or slums (Omigbodun et al., 2008; Swahn et al., 2012; Akkaya-Kalayci et al., 2015). Urban stresses tend to cause depression due to failure to make a living (Kinyanda et al., 2012).

Substance or alcohol abuse has also been observed to be a leading cause of suicide ideation or attempt (Ikealumba and Couper, 2006; Swahn et al., 2014; Sendagala et al., 2018; Nyundo et al., 2020). Substance or alcohol abuse is known to impair someone's judgment, making them engage in risky behavior such as suicidality (Swahn et al., 2013, 2014). Nyundo et al. (2020) observe that adolescents are likely to illegally use drugs because they think that drug use is a form a problem-solving.

While there are a number of studies that have examined suicide ideation and attempts in developing countries, few studies have examined suicide ideation and attempt among vulnerable adolescents living in urban areas or slums (Davaasambuu et al., 2017; Yildiz et al., 2019). This study fills this gap by exploring the risk factors associated with suicide ideation and attempt among vulnerable adolescents living in poor urban settlements in Kampala, Uganda. The results from these analyses can shed light on the dynamics and risk factors of suicide ideation or attempt among adolescents living on streets or in slums.

## Study Methods

The study was based on analysis of data collected in August 2020 as part of monitoring and evaluation of the project by UYDEL supporting vulnerable adolescents and youth. UYDEL aims at enhancing socio-economic transformation of vulnerable youth through advocacy, psychosocial, and skills development for self-reliance and reintegration. The data analyzed were collected from 219 adolescents (13–19 years) drawn from a list of beneficiaries receiving support from UYDEL.

UYDEL has six centers located in Kampala city (Bwaise, Nakawa, Nateete, Masooli, Nankulabye, and Kamwokya). UYDEL is a non-government organization (NGO) that works with adolescents and youths in the age group 10–24 years. These adolescents and youths are mostly vulnerable and at risk of exploitation and abuse. UYDEL's aim is to enhance the socio-economic transformation of vulnerable adolescents and youths through advocacy for their rights and skills development (Bukuluki et al., 2019; Uganda Youth Development Link, 2021). In 2020, UYDEL served 805 adolescents (Uganda Youth Development Link, 2021). All respondents for this study were recruited through UYDEL. We generated a sampling frame from all lists provided by the six UYDEL centers of adolescents (aged 13–19 years). Selection of eligible adolescents for interview followed systematic sampling. UYDEL provided contact(s) of all eligible adolescents that were selected for interview. Social workers reached out to all eligible adolescents using phone contacts provided by UYDEL and explained the purpose of the study. Adolescents who agreed to participate in the study provided the convenient time and venue for the interview with the social workers. All interviews were conducted at a time (August 2020) when restrictions on mobility due to COVID-19 had been lifted (Development Initiatives, 2020; Government of Uganda, 2020). During the interview process, all Standard Operating Procedures (SOPs) for collecting data during the COVID-19 pandemic as guided by the World Health Organization were followed (WHO, 2020b). The interviewer (social worker) and the interviewee (adolescent) wore face masks, adhered to social distancing guidelines and used sanitizers. Out of the estimated total sample size of 250 adolescents, only 219 adolescents responded to the interview—giving a response rate of 88%. Variables that were used in our analysis included socio-demographic characteristics (age, education, marital status, religion, employment status, self-reported HIV status, and residence status), having considered suicide in the past 4 months, attempted suicide in the past 6 months, alcohol consumption, drug and substance abuse, and psychological distress scores measured using the Kessler Psychological Distress Scale (K10). The scale considers 10 questions: feeling tired, nervous, nervous without calming down, hopeless, restless, restless without sitting still, depressed, feeling that everything is an effort, sad, and feeling worthless. Kessler's K10 question scale designed to measure the level of unspecified psychological distress has been used in clinical and population surveys (Kessler et al., 2003). Following previous studies (Mewton and Andrews, 2015; Matheson et al., 2016; Sweetland et al., 2018; Chen et al., 2020; Sopheab et al., 2020), we categorized adolescents with <20 score as low, 20–24 as moderate, 25–29 as high, and 30+ as very high or severe. The questions on suicidality included the following: (a) Have you ever had thoughts of killing yourself in the last 4 weeks? (b) Have you ever had thoughts of killing yourself in the last 1 week? (c) Have you tried killing yourself in the last 6 months?

Data analysis was performed using Stata software version 15.0 (Stata Corporation, College Station, TX, USA). We obtained frequencies and percentages of the different attributes. Poisson regression model was used to identify potential risk factors for suicidal ideation and suicidal attempt. Only factors with log-likelihood ratio test *p* < 0.05 in the bivariate model against the null model were included in the multivariable Poisson regression model.

Ethical considerations required in conducting research with adolescents as well as the public health measures for collecting data during COVID-19 were ensured. Permission to conduct the study was granted by the School of Social Sciences Research Ethics Committee at Makerere University (MAKSS REC 02.20.390). We sought consent from adolescents who are 18 years and above. However, we obtained consent (from parents or guardian) and assent from adolescents who were below 18 years at the time of the survey. Emancipated minors (adolescents living alone, married, mothers) provided oral consent to participate in the survey. The interview duration ranged from 45 min to 1 h. During the interview, social workers (who conducted interviews) offered counseling and referral for further management of any adolescents who exhibited suicide ideation and attempt.

## Results

### Sample Characteristics

Table 1 shows the distribution of respondents included in the study. Of the 219 adolescents interviewed, 68% were female, 27% were aged 13–15 years, 51% had at least some secondary school education, and 18% were still students. Among out-of-school adolescents, only 21% were employed (Table 1). About 7% of adolescents were living in a marital relationship and 83% reported to have family or friends whom they confide in when faced with social or psychosocial challenges. Furthermore, 25% reported to abuse alcohol or substances. There were no significant differences in the distribution of socio-demographic characteristics between the boys and girls. However, more boys than girls reported alcohol abuse (31.0 vs. 21.6%), drugs, and substance abuse (15.5 vs. 5.4%) (see Table 1).

**Table 1:** Sample demographics and behavioral characteristics.

	**Female**		**Male**		**Total**	
	***n***	**%**	***n***	**%**	***n***	**%**
**Age group**
13–15 years	48	32.4	11	15.5	59	26.9
16–19 years	100	67.6	60	84.5	160	73.1
**Residence status**
National	140	94.6	64	90.1	204	93.2
Refugee	8	5.4	7	9.9	15	6.8
**Education level**
Primary	75	50.7	32	45.1	107	48.9
Some secondary	73	49.3	39	54.9	112	51.1
**Religious affiliation**
Roman catholics	38	25.7	16	22.5	54	24.7
Anglicans	29	19.6	12	16.9	41	18.7
Pentecostal	42	28.4	24	33.8	66	30.1
Muslim	39	26.4	16	22.5	55	25.1
Other	0	0.0	3	4.2	3	1.4
**Marital status**
Married	11	7.4	4	5.6	15	6.8
Single	137	92.6	67	94.4	204	93.2
**HIV status (self-reported)**
Negative	91	61.5	50	70.4	141	64.4
Positive	3	2.0	0	0.0	3	1.4
Not sure	54	36.5	21	29.6	75	34.2
**Employment status**
Not employed	98	66.2	45	63.4	143	65.3
Employed	17	11.5	20	28.2	37	16.9
Student	33	22.3	6	8.5	39	17.8
**Alcohol abuse**
No	116	78.4	49	69.0	165	75.3
Yes	32	21.6	22	31.0	54	24.7
**Drug and substance abuse**
No	140	94.6	60	84.5	200	91.3
Yes	8	5.4	11	15.5	19	8.7
**Has a friend/family they can confide in**
No	20	13.5	17	23.9	37	16.9
Yes	128	86.5	54	76.1	182	83.1
**Psychological distress score (K10)**
<20 (Low)	43	29.1	24	33.8	67	30.6
20–24 (Moderate)	34	23.0	20	28.2	54	24.7
25–29 (High)	28	18.9	4	5.6	32	14.6
30+ (Very high/severe)	43	29.1	23	32.4	66	30.1
**Suicide ideation and attempt**
Suicide ideation in the past 4 weeks	50	33.8	17	23.9	67	30.6
Suicide ideation in the past 1 week	23	16.1	5	7.4	28	13.3
Suicide plan for suicide in the past 1 week	17	11.5	4	5.6	21	9.6
Ever attempted suicide	71	48.0	30	42.3	101	46.1
Attempted suicide in the last 6 months	35	23.6	18	25.4	53	24.2

### Prevalence of Suicidal Ideation and Attempts

Table 1 shows that suicidal attempt was reported by 46.1% (95% CI: 39.4, 53.0%) of the adolescents interviewed. Of the 101 youth who have ever attempted suicide, 46.5% did so for the first time within the last 2–6 months preceding the survey.

The prevalence of suicidal ideation was 30.6% (95% CI: 24.3, 36.0%) in the past 4 weeks and 13.3% (95% CI: 9.0, 18.6%) in the past 1 week preceding the survey. Over 75% of the youth who planned to commit suicide in the last 1 week preceding survey had a clear suicide plan. The prevalence of attempt within the past 6 months preceding the survey was 24.2% (95% CI: 18.7, 30.4%) (Table 1). The most stressful precursors of suicidal ideation or attempt included financial difficulties reported by 59.5% of the adolescents, family breakdown or violence reported by 39.4%, and trauma reported by 23.1%. Other precursors include loss of a loved one (22.6%), sexual relationship-related challenges (15.4%), and chronic pain or illness (10.8%). Seventy percent of the adolescents had some form of psychological distress (data not shown).

### Factors Associated With Suicidal Ideation and Attempt

The results in Table 2 indicate that suicidal ideation in the past 4 weeks was more common among the girls than boys (33 vs. 23%), adolescents who abuse alcohol or drugs (56 vs. 22%), with no friend or family member to confide in when faced with challenges (46 vs. 27%), or have some psychological distress. Education level, age, religious affiliation, and employment status had no influence on suicidal ideation among adolescents interviewed.

**Table 2:** Univariate analysis of factors associated with suicide ideation and suicide attempt.

		**Suicide ideation within the last 4 weeks**			**Suicide attempt within the last 6 months**		
	***N***	***n***	**%**	**PR (95% CI)**	***n***	**%**	**PR (95% CI)**
**Sex**
Female	148	49	33.3	1.00	35	23.6	1.00
Male	71	16	22.9	0.69 (0.39, 1.21)	18	25.4	1.07 (0.61, 1.89)
**Age group**
13–15 years	59	15	26.3	1.00	14	23.7	1.00
16–19 years	160	50	31.3	1.19 (0.67, 2.11)	39	24.4	1.03 (0.56, 1.89)
**Residence**
National	204	59	29.2	1.00	51	25.0	1.00
Refugee	15	6	40.0	1.37 (0.59, 3.17)	2	13.3	0.53 (0.13, 2.19)
**Education level**
Primary	102	29	28.4	1.00	27	26.5	1.00
Some secondary	117	36	31.3	1.1 (0.68, 1.80)	26	22.2	0.84 (0.49, 1.44)
**Religious affiliation**
Roman catholics	54	12	22.2	1.00	12	22.2	1.00
Anglicans	41	14	34.1	1.54 (0.71, 3.32)	12	29.3	1.32 (0.59, 2.93)
Pentecostal	66	22	33.8	1.52 (0.75, 3.08)	17	25.8	1.16 (0.55, 2.43)
Muslim	55	17	31.5	1.42 (0.68, 2.97)	12	21.8	0.98 (0.44, 2.19)
**Marital status**
Married	15	10	66.7	1.00	7	46.7	1.00
Single	204	55	27.2	0.41 (0.21, 0.80)^*^	46	22.5	0.48 (0.22, 1.07)
**Alcohol abuse**
No	165	35	21.5	1.00	22	13.3	1.00
Yes	54	30	55.6	2.59 (1.59, 4.21)^*^	31	57.4	4.31 (2.49, 7.44)^*^
**Drug and substance abuse**
No	200	53	26.8	1.00	46	23.0	1.00
Yes	19	12	63.2	2.36 (1.26, 4.42)^*^	7	36.8	1.6 (0.72, 3.55)
**Employment status**
Not employed	143	46	32.4	1.00	39	27.3	1.00
Employed	37	11	29.7	0.92 (0.48, 1.77)	8	21.6	0.79 (0.37, 1.70)
Student	39	8	21.1	0.65 (0.31, 1.38)	6	15.4	0.56 (0.24, 1.33)
**Psychological distress score**
<20 (Low)	67	5	7.7	1.00	2	3.0	1.00
20–24 (Moderate)	54	12	22.2	2.89 (1.02, 8.2)^*^	9	16.7	5.58 (1.21, 15.8)^*^
25–29 (High)	32	13	40.6	5.28 (1.88, 14.8)^*^	6	18.8	6.28 (1.27, 21.1)^*^
30+ (Very high/severe)	66	35	53.0	6.89 (2.7, 17.6)^*^	36	54.5	18.27 (4.4, 45.9)^*^
**Has a friend/family s/he can confide in**
No	37	17	45.9	1.00	17	45.9	1.00
Yes	182	48	26.7	0.58 (0.33, 1.01)	36	19.8	0.43 (0.24, 0.77)^*^
**Youth aware of psychosocial support**
No	65	31	48.4	1.00	23	35.4	1.00
Yes	154	34	22.2	0.46 (0.28, 0.75)^*^	30	19.5	0.55 (0.32, 0.95)^*^

*Note:*

* * = p <0.05*.

Results from the multivariable Poisson model (Table 3) indicate that the prevalence of suicidal ideation in the past 4 weeks was five times higher among those with severe psychological distress (PRR = 4.75; 95% CI: 1.72, 13.08). Suicidal ideation was lower among adolescents who knew where to obtain professional psychological care (PRR = 0.51; 95% CI: 0.30, 0.87).

**Table 3:** Multivariate analysis of factors associated with suicide ideation and suicide attempt.

	***N***	**Suicide ideation within the last 4 weeks**		**Suicide attempt within the last 6 months**	
		**Adjusted PR (95% CI)**	***p*-value**	**Adjusted PR (95% CI)**	***p*-value**
**Sex**
Female	148	1.00		1.00	
Male	71	0.69 (0.38, 1.27)	0.236	1.07 (0.58, 1.98)	0.826
**Age group**
13–15 years	59	1.00		1.00	
16–19 years	160	1.17 (0.64, 2.12)	0.617	0.81 (0.43, 1.54)	0.517
**Marital status**
Married	15	1.00		1.00	
Single	204	0.57 (0.27, 1.17)	0.125	0.85 (0.37, 1.94)	0.692
**Alcohol abuse/substances**
No	165				
Yes	54	1.42 (0.79, 2.56)	0.236	2.13 (1.13, 4.01)^*^	0.020
**Psychological distress score**
<20 (Low)	67	1.00		1.00	
20–24 (Moderate)	54	2.74 (0.96, 7.84)	0.061	4.72 (1.01, 12.03)^*^	0.049
25–29 (High)	32	5.08 (1.77, 14.6)^*^	0.003	5.62 (1.11, 18.4)^*^	0.037
30+ (Very high/severe)	66	4.75 (1.72, 13.08)^*^	0.003	11.8 (4.66, 32.37)^*^	0.001
**Has a friend/family s/he can confide in**
No	37	1.00		1.00	
Yes	182	1.16 (0.61, 2.2)	0.659	1.18 (0.6, 2.33)	0.634
**Youth aware of psychosocial support**
No	65	1.00		1.00	
Yes	154	0.51 (0.30, 0.87)^*^	0.013	0.8 (0.45, 1.43)	0.447

*Note:*

* * = p <0.05*.

Similar to suicidal ideation, prevalence of suicidal attempt in the past 6 months was higher among adolescents who abuse alcohol and drugs (57 vs. 13%) and those with any form of psychological distress. The prevalence of suicidal attempt was lower among adolescents with friends or family members to confide in when faced with challenges and those who knew where to obtain professional psychological care.

Table 3 shows that the prevalence of suicidal attempt was 5 times higher among respondents with moderate psychological distress (PR = 4.72; 95% CI: 1.01, 12.03) and over 10 times higher among adolescents with severe psychological distress (PR = 11.80; 95% CI: 4.66, 32.32). Adolescents who abuse drugs or substances had a higher prevalence of suicide attempts than those who do not (PR = 2.13; 95% CI: 1.13, 4.01).

## Discussion

The aim of this study was to assess suicidal ideations and attempts among vulnerable adolescents. In this study, suicide ideation was measured for the last 4 weeks prior to the survey while suicide attempt had a reference period of 6 months preceding the survey. Results indicate a high burden of suicidal ideation and attempt among vulnerable adolescents living in poor urban settlements in Kampala, Uganda. The prevalence of suicidal ideation and attempt among adolescents was 31 and 24%, respectively. Suicidal ideation was higher in comparison with a nationally representative youth survey in Uganda (22%) (Swahn et al., 2012), but similar to what was reported by Swahn et al. among adolescents in slums in Kampala in 2012 (31% in past 12 months) (Swahn et al., 2012). While there is likely to be a number of plausible reasons for such an observation, results from this study indicate that financial difficulties, living without a natal family or with broken families, and psychological distress are the probable reasons for a high burden of suicidal ideations and attempts.

Results from the multivariate analyses indicate that suicidal ideation was higher among adolescents with severe psychological distress, but suicide attempt was higher among adolescents with moderate or severe psychological distress. Suicide attempt was observed to be higher among drug or alcohol abusers while suicide ideation was lower among adolescents who knew where to obtain professional psychological care. Swahn et al. (2012) observed that the occurrence of suicidal ideation is largely associated with severe psychological stress. Psychological stress may result from losing a parent or someone to HIV/AIDS, stigma, discrimination, isolation, or not having social support (Swahn et al., 2012).

Previous research has shown that adolescents living on streets or in slums in urban areas experience financial difficulties and are likely to use alcohol and/or drugs, lack enough food, have poor quality of life, are lonely, or have a history of mental illness (Rudatsikira et al., 2007; Kinyanda et al., 2012; Swahn et al., 2012, 2014; Mugisha et al., 2016; Rukundo et al., 2016). These factors facilitate the occurrence of suicidal ideation or attempt. Other plausible reasons for suicidal ideation or attempt from previous research are depression, hopelessness, lack of social support, and failure to provide for the family (Rukundo et al., 2016).

Our results confirm earlier studies about substance abuse being a potential risk factor of suicidal attempt in sub-Saharan Africa (Sendagala et al., 2018). Drug or alcohol abuse has been found to impair judgment or reasoning (Asiki et al., 2011) and even lead to more risky behavior such as sexual relations and fights (Swahn et al., 2014). Results from eight sites in six countries (Ethiopia, Burkina Faso, Ghana, Nigeria, Tanzania, and Uganda) indicated a significant association between drug abuse and depression, which is a major determinant of suicidal ideation (Ikealumba and Couper, 2006; Nyundo et al., 2020). Adolescents may resort to substance abuse when they are experiencing psychological distress as a coping strategy (Nyundo et al., 2020). Nyundo et al. (2020) contend that the factors associated with suicidal ideation can be prevented. Given the findings reported in this study, we therefore join Nyundo's et al. (2020) in suggesting routine screening and counseling for early detection of psychological distress among vulnerable adolescents in urban settlements. Routine counseling among a high risky population group can help with devising early intervention strategies (Nyundo et al., 2020).

Adolescents with psychological disorders often do not seek or get the needed help, care, or counseling either because of failure to diagnose, financial constraints, or not being recognized as a health problem (Ssebunnya et al., 2019). Furthermore, efforts to prevent and reduce substance abuse among adolescents through social behavioral change communication and psychosocial support or education should be intensified. We add our voice to the call made by Mugisha et al. advocating for improved health services and policies to better manage psychosocial problems that can lead to suicide ideations or attempts (Mugisha et al., 2016) particularly in low- and middle-income countries.

## Conclusion

The findings we report in this study indicate that substance abuse and psychological stress are significant factors associated with suicide ideation and attempt among adolescents living in urban areas in Kampala, Uganda. We contend that substance abuse or psychological stress is likely to be triggered by challenges that come with living in poor urban settings. This study contributes to understanding of the risk factors of suicidal ideation and attempt among adolescents living in urban settlements in Kampala, Uganda.

### Limitations

Three main limitations emerge from the present study. First, data used in this study come from a cross-sectional survey that does not permit the analysis of the direction of causality. Therefore, results from the psychological distress score (K10) may be symptomatic since no further medical assessment was done. Second, the results we report in this study may suffer from selection bias, since all the adolescents we recruited through UYDEL are vulnerable. Third, although this study may provide context, the results may not be generalizable to other populations because of a relatively small sample size, and last, the results presented in this study may be influenced by the COVID-19 pandemic even though we did not directly investigate the effect of COVID-19 pandemic on what we studied. Despite the above limitations, this study addresses an important topic that is bridging knowledge gap in understanding of the risk factors of suicide ideation and suicide attempt among vulnerable adolescents in urban settlements. Furthermore, the findings presented in this study provide evidence that can guide the design of prevention strategies and therapeutic interventions targeting vulnerable adolescents living in poor urban settlements similar to those in Kampala, Uganda.

### Recommendation

Given the challenges associated with the COVID-19 pandemic, future studies should investigate the effects of COVID-19 on suicidal ideation and attempt.

## Data Availability Statement

The original contributions presented in the study are included in the article/supplementary material, further inquiries can be directed to the corresponding author/s.

## Ethics Statement

The studies involving human participants were reviewed and approved by School of Social Sciences Research Ethics Committee at Makerere University. Written informed consent to participate in this study was provided by the participants' legal guardian/next of kin.

## Author Contributions

PB contributed to study design, data analysis, manuscript writing, and discussion. SW conducted data analysis, manuscript writing, and discussion of results. PK contributed to discussion and manuscript writing. SB contributed to study design and review of study tools. RK contributed to review of study tools and manuscript.

## Conflict of Interest

The authors declare that the research was conducted in the absence of any commercial or financial relationships that could be construed as a potential conflict of interest.

## Abbreviations

NGO: Non-Government Organization
SOPs: Standard Operating Procedures
UYDEL: Uganda Youth Development Link
WHO: World Health Organization.

## References

[B1] Akkaya-Kalayci T. Popow C. Winkler D. Bingöl R. H. Demir T. Özlü Z. (2015). The impact of migration and culture on suicide attempts of children and adolescents living in Istanbul. Int. J. Psychiatry Clin. Pract. 19, 32–39. 10.3109/13651501.2014.961929 25195766

[B2] Ammerman B. A. Burke T. A. Jacobucci R. McClure K. (2021). Preliminary investigation of the association between COVID-19 and suicidal thoughts and behaviors in the U.S. J. Psychiatr. Res. 134, 32–38. 10.1016/j.jpsychires.2020.12.037 33360222

[B3] Asiki G. Mpendo J. Abaasa A. Agaba C. Nanvubya A. Nielsen L.. (2011). HIV and syphilis prevalence and associated risk factors among fishing communities of Lake Victoria, Uganda. Sex. Transm. Infect. 87, 511–515. 10.1136/sti.2010.046805 21835763

[B4] Bryan C. J. Bryan A. O. Baker J. C. (2020). Associations among state-level physical distancing measures and suicidal thoughts and behaviors among U.S. adults during the early COVID-19 pandemic. Suicide Life-Threat. Behav. 50, 1223–1229. 10.1111/sltb.12653 32589801 PMC7362130

[B5] Bukuluki P. M. Kamya S. Kasirye R. Nabulya A. (2019). Facilitating the transition of adolescents and emerging adults from care into employment in Kampala, Uganda: a case study of Uganda Youth Development Link. Emerg. Adulthood 8, 35–44. 10.1177/2167696819833592

[B6] Carr D. (2012). Death and dying in the contemporary United States: what are the psychological implications of anticipated death? Soc. Personal. Psychol. Compass 6, 184–195. 10.1111/j.1751-9004.2011.00416.x

[B7] Chen Y. Chen S. Arayasirikul S. Wilson E. McFarland W. Lu J.. (2020). A cross-sectional study of mental health, suicidal ideation and suicide attempt among transgender women in Jiangsu province, China. J. Affect. Disord. 277, 869–874. 10.1016/j.jad.2020.09.002 33065828 PMC8064745

[B8] Culbreth R. Masyn K. E. Swahn M. H. Self-Brown S. Kasirye R. (2021). The interrelationships of child maltreatment, alcohol use, and suicidal ideation among youth living in the slums of Kampala, Uganda. Child Abuse Negl. 112:104904. 10.1016/j.chiabu.2020.104904 33385928 PMC7855690

[B9] Culbreth R. Swahn M. H. Ndetei D. Ametewee L. Kasirye R. (2018). Suicidal ideation among youth living in the slums of Kampala, Uganda. Int. J. Environ. Res. Public Health 15:298. 10.3390/ijerph15020298 29425129 PMC5858367

[B10] Davaasambuu S. Batbaatar S. Witte S. Hamid P. Oquendo M. A. Kleinman M.. (2017). Suicidal plans and attempts among adolescents in Mongolia. Crisis 38, 330–343. 10.1027/0227-5910/a000447 28228061

[B11] Development Initiatives. (2020). Socioeconomic Impact of Covid-19 in Uganda: How has the Government Allocated Public Expenditure for FY2020/21? Kampala. Available online at: https://devinit.org/resources/socioeconomic-impact-of-covid-19-in-uganda/#downloads (accessed February 14, 2021).

[B12] Gjertsen F. Bruzzone S. Griffiths C. E. (2019). Burden of suicide presented as one of the leading causes of death: uncover facts or misrepresent statistics? J. Glob. Health 9:010401. 10.7189/jogh.09.010401 30479749 PMC6237205

[B13] Government of Uganda (2020). Update on COVID-19 Response in Uganda. Kampala: Government of Uganda. Available online at: https://www.health.go.ug/cause/update-on-covid-19-response-in-uganda-22-december-2020/ (accessed February 14, 2021).

[B14] Hottes T. S. Ferlatte O. Gesink D. (2015). Suicide and HIV as leading causes of death among gay and bisexual men: a comparison of estimated mortality and published research. Crit. Public Health 25, 513–526. 10.1080/09581596.2014.946887

[B15] Ikealumba N. Couper I. (2006). Suicide and attempted suicide: the Rehoboth experience. Rural Remote Health 6, 1–9. 17073530

[B16] Kessler R. C. Barker P. R. Colpe L. J. Epstein J. F. Gfroerer J. C. Hiripi E.. (2003). Screening for serious mental illness in the general population. Arch. Gen. Psychiatry. 60, 184–189. 10.1001/archpsyc.60.2.184 12578436

[B17] Kinyanda E. Hoskins S. Nakku J. Nawaz S. Patel V. (2012). The prevalence and characteristics of suicidality in HIV/AIDS as seen in an African population in Entebbe district, Uganda. BMC Psychiatry 12, 1–9. 10.1186/1471-244X-12-63 22713589 PMC3395571

[B18] Luxton D. D. June J. D. Fairall J. M. (2012). Social media and suicide: a public health perspective. Am. J. Public Health 102, S195–S200. 10.2105/AJPH.2011.300608 22401525 PMC3477910

[B19] Mamun M. A. Griffiths M. D. (2020). First COVID-19 suicide case in Bangladesh due to fear of COVID-19 and xenophobia: possible suicide prevention strategies. Asian J. Psychiatr. 51, 102073–102073. 10.1016/j.ajp.2020.102073 32278889 PMC7139250

[B20] Matheson K. M. Barrett T. Landine J. McLuckie A. Soh N. L.-W. Walter G. (2016). Experiences of psychological distress and sources of stress and support during medical training: a survey of medical students. Acad. Psychiatry 40, 63–68. 10.1007/s40596-015-0395-9 26223316

[B21] Mewton L. Andrews G. (2015). Cognitive behaviour therapy via the internet for depression: a useful strategy to reduce suicidal ideation. J. Affect. Disord. 170, 78–84. 10.1016/j.jad.2014.08.038 25233243

[B22] Mugisha J. Muyinda H. Kagee A. Wandiembe P. Mpugu S. K. Vancampfort D.. (2016). Prevalence of suicidal ideation and attempt: associations with psychiatric disorders and HIV/AIDS in post-conflict Northern Uganda. Africa Health Sci. 16, 1027–1035. 10.4314/ahs.v16i4.20 28479896 PMC5398450

[B23] Nyundo A. Manu A. Regan M. Ismail A. Chukwu A. Dessie Y.. (2020). Factors associated with depressive symptoms and suicidal ideation and behaviours amongst sub-Saharan African adolescents aged 10-19 years: cross-sectional study. Trop. Med. Int. Health 25, 54–69. 10.1111/tmi.13336 31698526

[B24] Omigbodun O. Dogra N. Esan O. Adedokun B. (2008). Prevalence and correlates of suicidal behaviour among adolescents in Southwest Nigeria. Int. J. Soc. Psychiatry 54, 34–46. 10.1177/0020764007078360 18309757

[B25] Page R. M. West J. H. (2011). Suicide ideation and psychosocial distress in Sub-Saharan African youth. Am. J. Health Behav. 35, 129–141. 10.5993/AJHB.35.2.1 21204676

[B26] Quarshie E. N.-B. Waterman M. G. House A. O. (2020). Self-harm with suicidal and non-suicidal intent in young people in sub-Saharan Africa: a systematic review. BMC Psychiatry 20, 1–26. 10.1186/s12888-020-02587-z 32408896 PMC7222461

[B27] Rudatsikira E. Muula A. S. Siziya S. Twa-Twa J. (2007). Suicidal ideation and associated factors among school-going adolescents in rural Uganda. BMC Psychiatry 7, 1–6. 10.1186/1471-244X-7-67 18034906 PMC2219990

[B28] Rukundo G. Z. Mishara B. L. Kinyanda E. (2016). Burden of Suicidal Ideation and Attempt among Persons Living with HIV and AIDS in Semiurban Uganda. AIDS Res. Treat, 2016, 1–10. 10.1155/2016/3015468 27073694 PMC4814631

[B29] Sendagala S. Ssenkusu J. M. Lubwama G. W. Bagenda D. Muyonga M. Hladik W. (2018). Suicide ideation, school absenteeism and physical violence among secondary school students in Kampala, Uganda. Int. J. Publ. Health Sci. 7, 293–302. 10.11591/ijphs.v7i4.14589

[B30] Sher L. (2020). The impact of the COVID-19 pandemic on suicide rates. QJM Int. J. Med. 113, 707–712. 10.1093/qjmed/hcaa202 32539153 PMC7313777

[B31] Sopheab H. Suy S. Chhea C. Chhit S. Mun P. Bui T. C. (2020). Psychological distress among Cambodian people who use drugs. Drug Alcohol Rev. 39, 66–70. 10.1111/dar.13000 31646699

[B32] Ssebunnya J. Medhin G. Kengere S. Kigozi F. Nakku J. L. C. (2019). Prevalence, correlates and help-seeking behaviour for depressive symptoms in rural Uganda: a population-based survey. Global Mental Health 6, 1–10. 10.1017/gmh.2019.25 31807311 PMC6880248

[B33] Swahn M. H. Haberlen M. Palmier J. B. Kasirye R. (2014). Alcohol and drug use and other high-risk behaviors among youth in the slums of Kampala, Uganda: perceptions and contexts obtained through focus groups. Int. J. Alcohol Drug Res. 3, 289–305. 10.7895/ijadr.v3i4.171

[B34] Swahn M. H. Palmier J. B. Kasirye R. (2013). Alcohol exposures, alcohol marketing, and their associations with problem drinking and drunkenness among youth living in the slums of Kampala, Uganda. ISRN Public Health 2013, 1–9. 10.1155/2013/948675

[B35] Swahn M. H. Palmier J. B. Kasirye R. Yao H. (2012). Correlates of suicide ideation and attempt among youth living in the Slums of Kampala. Int. J. Environ. Res. Public Health 9, 596–609. 10.3390/ijerph9020596 22470312 PMC3315266

[B36] Sweetland A. C. Norcini Pala A. Mootz J. Kao J. C.-W. Carlson C. Oquendo M. A.. (2018). Food insecurity, mental distress and suicidal ideation in rural Africa: evidence from Nigeria, Uganda and Ghana. Int. J. Soc. Psychiatry 65, 20–27. 10.1177/0020764018814274 30479180 PMC6386592

[B37] Tasnim R. Islam M. S. Sujan M. S. H. Sikder M. T. Potenza M. N. (2020). Suicidal ideation among Bangladeshi university students early during the COVID-19 pandemic: prevalence estimates and correlates. Child. Youth Serv. Rev. 119:105703. 10.1016/j.childyouth.2020.105703 33204046 PMC7654299

[B38] Uganda Youth Development Link (2021). Uganda Youth Development Link. Available online at: http://www.uydel.org/ (accessed February 11, 2021).

[B39] WHO (2020a). Adolescent Health Epidemiology. Geneva: WHO. Available online at: http://www.who.int/maternal_child_adolescent/epidemiology/adolescence/en/ (accessed December 17, 2020).

[B40] WHO (2020b). Ethical Standards for Research During Public Health Emergencies: Distilling Existing Guidance to Support COVID-19 R&D. Geneva: WHO. Available online at: https://www.who.int/publications/i/item/WHO-RFH-20.1 (accessed February 7, 2021).

[B41] Yildiz M. Orak U. Walker M. H. Solakoglu O. (2019). Suicide contagion, gender, and suicide attempts among adolescents. Death Stud. 43, 365–371. 10.1080/07481187.2018.1478914 29920166

